# Understanding Barriers and Enablers to State Action on Salt: Analysis of Stakeholder Perceptions of the VicHealth Salt Reduction Partnership

**DOI:** 10.3390/nu11010184

**Published:** 2019-01-16

**Authors:** Briar McKenzie, Kathy Trieu, Carley A. Grimes, Jenny Reimers, Jacqui Webster

**Affiliations:** 1 The George Institute for Global Health, University of New South Wales, Sydney, NSW 2042, Australia; ktrieu@georgeinstitute.org.au (K.T.); jwebster@georgeinstitute.org.au (J.W.); 2 Sydney School of Public Health, Faculty of Medicine, The University of Sydney, Sydney, NSW 2006, Australia; 3 Institute for Physical Activity and Nutrition (IPAN), School of Exercise and Nutrition Sciences, Deakin University, Geelong, VIC 3220, Australia; carley.grimes@deakin.edu.au; 4 The Victorian Health Promotion Foundation (VicHealth), Melbourne VIC 3053, Australia; jreimers@vichealth.vic.gov.au

**Keywords:** sodium reduction, public health nutrition, hypertension, stakeholder perceptions, disease prevention, population interventions

## Abstract

The Victorian Salt Reduction Partnership (VicSalt Partnership) was launched in 2015, bringing together health and research organisations to develop an action plan for salt reduction interventions at a state level. A comprehensive evaluation was designed to assess the impact of the resulting four-year intervention strategy. As part of the process evaluation, semi-structured interviews were undertaken with stakeholders in March–May 2017, to understand perceived barriers and enablers to effective strategy implementation. Data were coded in relation to the key topic areas of the interviews with an inductive method used to analyse themes within topics. Seventeen stakeholders were contacted, 14 completed an interview; five from state government or statutory agencies, four from non-government funded organisations, four from research organisations and one from the food industry. Twelve were members of the VicSalt Partnership and two were informal collaborators. Most stakeholders viewed the VicSalt Partnership as a positive example of working collaboratively, and said this was essential for raising awareness of the importance of salt reduction with consumers, the food industry, and the government. Challenges relating to engaging the food industry and federal government through a state-led initiative were identified. New approaches to overcome this, such as forming clear “asks” to government and committing industry to “pledges” on reformulation were suggested. Stakeholder interviews and qualitative analysis have provided a range of important insights into barriers and enablers, many of which have already been used to strengthen intervention implementation. The evaluation of the VicSalt Partnership is ongoing and the program is expected to provide a wealth of lessons for state-led interventions to reduce salt intake in Australia and globally.

## 1. Introduction

Australians are eating almost twice the recommended amount of salt [1], a problem given the strong relationship between salt intake, blood pressure and cardiovascular disease risk [2,3]. Australia is not alone with this problem, globally population salt intake is high, putting people at increased risk of morbidity and mortality [4,5]. As such, the World Health Organisation and United Nations have made population salt reduction a priority, as part of the commitment to reduce non-communicable disease by 25% by the year 2025 [6]. This goal is incentivised by salt reduction initiatives being one of the most cost-effective prevention factors for cardiovascular disease [7]. The Australian government agreed to this target [6] yet currently there is no comprehensive federal government strategy to reduce population salt intake.

Victoria is the most densely populated state in Australia with approximately 6.3 million people living in this south-eastern region [8] of which 25% are affected by high blood pressure (2010) [9]. Additionally, Victoria is the centre for many large food companies and, therefore, is an opportunistic area to focus salt-reduction efforts. In 2014 a review was conducted of salt reduction initiatives at a state level, called *The case for state action on salt reduction* [10]. State-level action provides the opportunity to “pilot” interventions and support action on a national level. For example, in the United States the New York City Health Department led requirements for sodium warning rules across chain restaurants and initiated the National Salt and Sugar Reduction Initiative [11], and historically in Australia, state-level intervention was successful in showing the effectiveness of tobacco control measures [10,12]. Given similar intakes and sources of salt across Australia, it is hypothesised that salt-reduction efforts that have proven effective in Victoria could be transferable to other states [5,10]. The Victorian Health Promotion Foundation (VicHealth) brought together a taskforce to consider the evidence review and to formulate an action plan for Victoria, through VicHealth investment. This led to VicHealth establishing a strategic Salt Reduction Partnership Group (from now on referred to as “the VicSalt Partnership”) at the end of 2014, launched in May 2015. The VicSalt Partnership consists of nine state and national organisations including; VicHealth, the George Institute for Global Health, the Heart Foundation Victoria, Deakin University’s Institute for Physical Activity and Nutrition, National Stroke Foundation, Kidney Health Australia, Baker Heart and Diabetes Institute, the High Blood Pressure Research Council and the Victorian Department of Health and Human Services [13]. The strategic VicSalt Partnership was brought together in order to coordinate salt reduction strategies at the state level. Collectively, the partnership released the *State of Salt*, a summary of the evidence based on *The case for state action on salt reduction*, with an action plan for salt reduction in Victoria [9]. VicHealth and The Heart Foundation (Victoria) are leading intervention implementation in consultation with the larger VicSalt Partnership group and additional informal collaboration with other organisations, including food industry representatives [14].

**Figure 1:**
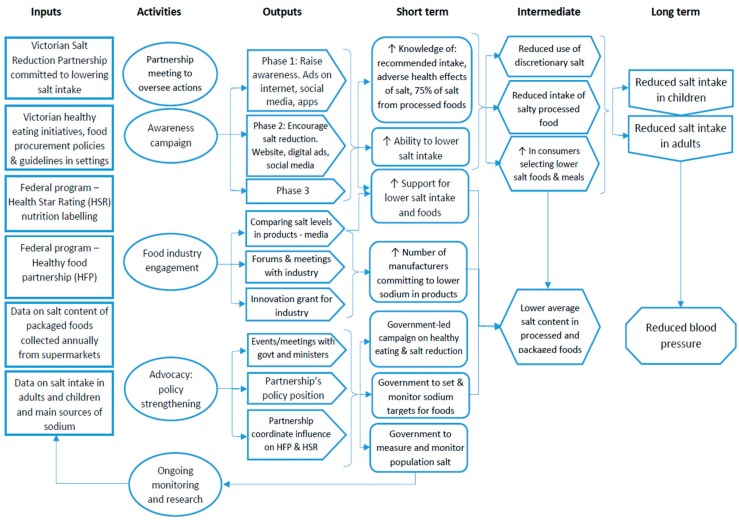
Logic model of the VicSalt Partnership program of interventions. Source: Trieu et al. 2018 [14].

The overarching goal of the VicSalt Partnership was to achieve a 1 g/day reduction in average salt intake for both adults and children [13]. The initial timeline was to reach this target by June 2018 [13], however with additional funding granted by VicHealth to extend planned intervention activities, the end-point for assessment was moved to the end of 2019 [14]. The intervention strategies were informed by the evidence review of state-level initiatives to reduce salt, prepared by the WHO Collaborating Centre for Population Salt Reduction at the George Institute [9]. This review formed the basis of the State of Salt report, launched in May 2015 and detailing the salt-reduction initiatives to be implemented in Victoria, aiming to: build strong partnerships, raise consumer awareness, generate public debate, strengthen existing federal and state policies, develop innovative approaches to engage the food industry, and monitor and evaluate effectiveness [9]. The activities to achieve the five aims are depicted in the program’s logic model by Trieu et al. [14], Figure 1. For example “raising consumer awareness” is made up of three phases of targeted messaging communicated through a range of mass and digital media. The interventions to engage the food industry in reformulation include releasing media reports of food products that have the lowest and highest salt content within their category and the introduction of an innovation grant to aid small-to-medium enterprises in food reformulation efforts [15].

In 2015 the George Institute, in partnership with other members of the VicHealth Salt Partnership, was awarded a National Health and Medical Research Council of Australia Partnership grant to evaluate the interventions over a four-year period [14]. Components of the evaluation include: collection of 24-h urine in children [16] and adults to assess salt intake at baseline and three years with concurrent collection of self-reported dietary information and repeated consumer surveys to assess salt related knowledge, attitudes and behaviours [17,18]; regular collection of cost data; annual surveys and impact assessment of the consumer campaigns; and interim analysis of stakeholder perceptions. This process allows for the assessment of each step in the intervention pathway and for adjustments to be made during intervention implementation.

As part of the process evaluation, interviews were conducted with key stakeholders of the VicSalt Partnership from March–May 2017, at the early stages of intervention implementation [14]. The aim was to understand the context, including stakeholder perceptions of likely barriers, enablers and opportunities, relating to intervention implementation, as well as the appropriateness of the intervention approach and likely impact.

## 2. Materials and Methods

The interim stakeholder analysis was undertaken as part of a broader process evaluation of the VicSalt Partnership focusing on the context, adoption, reach, dose, implementation, effectiveness, and cost of the interventions [14,19]. In particular, the stakeholder analysis looked at gaining information relevant to the context, adoption, and effectiveness of interventions [20]. VicHealth provided a list of 17 potential interviewees to approach. The list included key personnel at VicHealth and from the other VicSalt Partnership organisations, along with additional informal collaborators who had been involved in initial salt-reduction consultations. At the beginning of March 2017 e-mails were sent to all on the contact list, with an information sheet and consent form. Confirmation of willingness to participate was through return e-mail, arranged interview time, and returned signed consent form. Interviews were conducted from March till May 2017 either in person or through Skype. The interviews were conducted by a lead researcher on the project (JW) and witnessed by a second researcher (BM), who was not previously involved on the project. Interviews followed a semi-structured questionnaire format with questions focusing on the main action areas outlined in the State of Salt; establishing strategic partnerships, generating public debate, raising consumer awareness, innovative approaches to dealing with industry and policy (see Table S1 for the full questionnaire) [9]. Specifically, questions on stakeholder’s views on barriers and enablers for each of the main action areas were asked, with probing questions to gain more in-depth answers where appropriate. A semi-structured approach allowed for flexibility to discuss other factors that may be affecting the partnership or interventions more broadly.

Permission to record interviews was sought. Audio interviews were manually transcribed and thematically analysed. The analysis was framed on the five key VicSalt Partnership actions (establishing strategic partnerships, generating public debate, raising consumer awareness, innovative approaches to dealing with industry and policy), focusing on identifying stakeholder perceived enablers, barriers and suggested approaches within each action area. Further themes within, and separate to, these topics were identified through an inductive method requiring line-by-line analysis of the transcripts [21]. The *NVivo analytical software system* was used for data management [19].

Preliminary data from these interviews where fed back to the VicSalt Partnership in June 2017. Actions taken, in the form of published publically available materials, following this feedback were reviewed and are reported on in the results.

This study was conducted according to the guidelines laid down in the Declaration of Helsinki and all procedures involving human subjects were approved by the University of Sydney Human Ethics Research Committee (2016/770). Written informed consent was obtained from all subjects.

## 3. Results

Out of the 17 organisations approached, 15 stakeholders agreed to be interviewed and 14 completed the interview process. Two stakeholders did not respond, one agreed yet changed jobs before the interview took place and could not complete an interview in the given timeframe. Stakeholders included were from state government or a statutory agency (SGSA), non-government organisations, food industry and research organisations; 12 were members of the VicSalt Partnership (Table 1). Interviews took an average of 45 min resulting in approximately six pages of transcribed audio per interview.

In total, 310 quotes were taken from the transcripts. The issue that created the most discussion within the interviews was regarding engagement with food industry (114 quotes across the 14 transcripts). The topic that resulted in the least amount of discussion was “generating public debate” (22 quotes). Key themes from the interviews are reported below, with additional quotes provided in Table S2.

The strength of the VicSalt Partnership group, both in terms of the people represented on the partnership, the partnership leadership and the clarity of program objectives was identified as an important enabling factor. The VicSalt Partnership was positively reflected upon as a “coalition of the willing”, with most interviewees identifying that the key experts needed to guide change are around the table. Most interviewees observed that the VicSalt Partnership strengthens the voice of the individual members, meaning they could gain more traction than if each member were to advocate for salt reduction in isolation.

*“I think we have absolutely pulled around the table the people who have skills and expertise, and could input in a way that could add such value”* (SGSA, member)

The need for a more strategic approach to food industry interventions and engagement in reformulation was seen as a key challenge and represented an area where there was still a lack of focus for the majority of the stakeholders interviewed. Interviewees were unclear of the goal of the innovative approaches with food industry action area, or what had been done in terms of engaging with industry. Most agreed that industry needed to be involved and targeted for salt reduction. Some stated that the process had been “too slow” and lacking direction. While some contact had been made, such as an industry roundtable with small-to-medium food business and case studies of Coles and Subway who have both made salt reduction efforts, it was not considered “enough” in the two-year timeframe. Several respondents mentioned that the effectiveness of the original focus in targeting small and medium-sized local industry would have been limited in impacting salt levels of foods given that majority of the food supply comes from the large, national-level manufacturers. However, it was recognised that working with national or international companies creates a further challenge for a state-driven initiative.

*“…the time that it takes to engage industry and develop relationships to achieve progress seems to be a much slower pace than we anticipated, again just getting it on the radar of industry”* (SGSA, member)

*“Just identifying Victoria-based companies is a bit challenging when you start talking food products, and ones that have large market share are not necessarily just going to stick to Victoria”* (NGO, member)

Many mentioned other ways industry might be involved such as through pledges or further case studies, with a view to engaging positively. Some interviewees suggested that industry needs to be given the “how” along with the “why” and have a form of “carrot and stick” motivation to change. An example of such an approach was through the use of salt-reduction targets and pledges from the food industry to meet these targets.

*“…being able to feed back some of the positive stories from industry to consumers to say these companies are doing the right thing, they have reduced their salt and are providing healthier food for your families”* (SGSA, member)

The need for stronger involvement of government and use of policies at a state and federal level was a strong focus of many of the discussions. At a state level several interviewees acknowledged that there are existing state healthy eating policies, *Healthy Choices* [22], and that it is important to work in line with these policies. However, others suggested that this context does not provide a strong enough message on the importance of salt reduction;

*“Victorian policy is a much broader healthy eating policy and healthy eating guidelines… I think it’s more about the fact that salt reduction is part of that and inherent within it but it is not the focus…”* (SGSA, member)

Some interviewees alluded to the fact that little could be done to reduce salt in the food supply without federal-level intervention. Several people said the federal government’s Healthy Food Partnership (HFP) [23], was currently lacking the driving force that population salt reduction needs, and that engaging with industry through the VicSalt Partnership was challenging because national salt-level targets for processed foods had yet to be agreed. On the other hand, the VicSalt Partnerships links to the HFP food reformulation group was seen as an enabling factor, giving the VicSalt Partnership insight into national plans and a platform to express their views. Some interviewees suggested that the industry engagement and awareness raising already being achieved by the VicSalt Partnership would facilitate stronger action at a national level, by demonstrating what could be achieved. Overall, the interviewees suggested that a federal government-level targeted national food and nutrition strategy was required to support state-level engagement with industry to reduce salt. One interviewee suggested this would entail regular comprehensive monitoring of dietary intakes, strategies to address inequalities in access to food and actions to address marketing of foods to children, factors not currently included in the HFP. The approach to achieve a national nutrition strategy was suggested through increasing advocacy of salt as a key health issue at both the state and federal level.

*“A lot of the things we are talking about are national or multi-national issues so I think that tension of state organisations trying to influence what is a national or a global environmental issues has probably got some question marks on it”* (SGSA, member)

*“My awareness of the federal government is that it’s predominately investing in the healthy food partnership, I’m not aware that they are doing much in the way of a coherent national nutrition policy, which I think is what many of us would have hoped for”* (Research, member)

The need for more strategic and bolder messaging in a crowded nutrition space was emphasised by most interviewees; *“It’s not just “eat less salt” and smile. This is a serious health risk*” (Research, member). Interviewees agreed that messaging to date has not been strong enough to stand out in the current *“crowded nutrition space”* (SGSA, member). The first consumer campaign, *Don’t Trust Your Taste Buds*, was seen as an achievement given it achieved wide reach through social media, public relations and paid media. Yet stakeholders felt it could have created more “waves” and said the time period was too short (running from May–June 2016). Almost all interviewees compared the salt campaign to the impact that the “sugar plight” has had over the past years, with a huge consumer drive behind it and said we needed to do the same for salt.

*“Another observation I would make around the nutrition landscape is what a crowded space it is, and things like sugar have a much higher profile… its getting the messages out there in what is a very busy space for health messages, particularly food messages”* (SGSA, member)

While the primary focus of the discussions was on what could be done rather than what had been done, there was agreement that the VicSalt Partnership had made substantial progress over the past two years. For example, in establishing and commencing work on multiple action plans corresponding to each arm of the intervention and in securing research funding to undertake a comprehensive process evaluation. Furthermore, actions taken following the interviews have been summarised in Table 2 [15,24,25,26,27].

## 4. Discussion

This is the first state-level partnership in Australia to address population salt intake and builds on the previous work of the Australian Division of World Action on Salt and Health (AWASH) [28]. This interim analysis of stakeholder perceptions of the partnership has highlighted strengths as well as identifying areas for enhancement. Whilst the VicSalt Partnership was seen as a positive initiative, the majority of interviewees expressed a need for clearer engagement with industry, stronger involvement of government, and more strategic and hard-hitting messaging to consumers. This feedback was taken on board by the VicSalt Partnership group and has been used to strengthen the intervention implementation [29].

The benefit of the VicSalt Partnership in working collaboratively to amplify messages was seen as a key strength of the initiative. Working collaboratively has been identified as instrumental to effectiveness in other salt-reduction strategies. The AWASH 2007 *Drop the Salt!* campaign was a collaboration between non-government organisations, health/medical and food industry organisations [30]. This campaign influenced the federal government in relation to the establishment of the Food and Health Dialogue (FHD) which preceded the current Healthy Food Partnership [31]. The FHD established voluntary salt content targets for 10 packaged processed foods running from 2009–2015, with subsequent evaluation showing moderate reduction in salt content [30,32]. Likewise, Castronuovo et al. [33] identified stakeholder collaboration as a strength of a successful voluntary initiative to reduce salt in processed foods in Argentina.

Reducing the consumption and production of processed packaged foods is crucial to improving population health [34,35], however, food reformulation is fundamental to population salt reduction in Australia given that the majority (~75–80%) of dietary salt comes from processed packaged foods [5]. Engaging the food industry to reformulate foods has proven effective in other countries with similar diets, with salt content targets aiding effectiveness in many [36,37]. This stakeholder analysis has revealed perceptions about the barriers to engagement with industry at a state level, and further reinforced the need for federal government-led action on salt. The use of salt-reduction “pledges” from industry and the promotion of previously agreed voluntary targets were discussed by interviewees. The Public Health Responsibility Deal in England [38] provides an example, and a wealth of lessons, on the use of voluntary pledges. The Responsibility Deal placed responsibility on the food industry to improve nutrition, with the resulting “public-private partnership” committee being 95% from the private food sector [39]. For interventions related to the salt-reduction pledges, 46% of organisations had “reformulation” as part of their delivery plans; however, evaluation by Knai et al. [39] found that none of these reformulation plans were motivated by the Responsibility Deal, acknowledging that evaluation of pledges was difficult given the limited requirement for reporting on plans and progress. Further evaluation of the Responsibility Deal, using a systems approach, raised questions on the partnership between the government and food industry, suggesting the strong leadership by industry limited the scope of interventions and compliance to voluntary targets [40]. These concerns are also discussed by Scrinis [35], commenting on voluntary reformulation allowing industry to modify within a narrow range, whereas a stronger regulatory approach, for example through government mandated salt content targets, increases the accountability of industry to make greater reductions. Most interviewees agreed that national policies to enforce salt-reduction targets would provide the strongest message and enabling factor to engage the food industry. Steps towards gaining national-level support from the federal government have been made following these interviews including a call to action to government based on the VicSalt Partnership work, with three asks: to set and monitor targets to reduce salt in identified food categories, measure and monitor changes in population salt intake, and deliver a national healthy eating campaign, which includes a focus on the importance of reducing salt consumption [26].

An enabler to the VicSalt Partnership’s influence was identified in having a member of the partnership on the current federal government’s Healthy Food Partnership (HFP) initiative. This initiative was established in 2016, with reference to provide “collective, voluntary action between government, the public health sector and the food industry to improve diets of Australians” [23]. The reformulation working group is currently establishing salt reduction targets, which were released for public consultation in September 2018 [41]. The VicSalt Partnership can support national action through advocacy and engagement with industry. Involvement with industry has increased significantly since the interviews, particularly as a result of the partnership’s work to highlight the varying salt content of products in different food categories. In July 2017 the *Unpack the Salt* campaign was launched by VicHealth and Heart Foundation Victoria, building on the *Don’t Trust Your Taste Buds* campaign [42]. *Unpack the Salt* aims to raise awareness of the high levels of salt contained in processed packaged foods. Within this campaign six product category reports have been conducted focusing on; bread, cooking sauces, ready meals, dips and crackers, sausages (processed meat) and Asian-style sauces [27]. These reports are formed from the analysis of routinely collected nutrient composition data from nutrition content labels on products in four main Australian supermarket chains. The findings have shown excessive levels of salt in all product categories, with large intra-category variance, calling out products that are excessively high in salt. Each report gained national media attention and incentivised industry engagement with the Heart Foundation, driving consumer awareness and building momentum to support reformulation. A similar approach of highlighting the wide variation of salt levels in processed foods was part of an effective salt-reduction strategy in the United Kingdom, resulting in a 20–40% reduction in salt levels in foods and a 15% reduction in population salt intake [43]. While this is more of a “stick” approach, endorsing pledges and having open discussions with industry will provide support and motivation for reformulation. Building on this, it is expected that the VicSalt Partnership’s engagement with industry on reformulation will enhance and support the HFP’s program of work, promoting the salt-reduction targets when they are released.

Health and nutrition-focused state government programs have the potential to influence population salt intake and, therefore, are an integral part of the VicSalt Partnership. The Victorian government’s “*Healthy Choices*” [22] focuses on: hospitals and health services, workplaces, sports and recreation centres and parks, providing guidelines for organisations to create their own healthy eating policies. While schools, workplaces and hospitals have been identified as key settings to target salt-reduction interventions [44], the majority of the interviewees were unclear whether “*Healthy Choices*” currently has the capacity to impact on salt intake, due to the general healthy eating focus. A role for the VicSalt Partnership in line with the state policy could be in supporting the monitoring and evaluation of “*Healthy Choices*” for salt-reduction effectiveness.

Our stakeholder analysis highlighted the importance of strong messaging to ensure that salt is seen in the crowded nutrition space, particularly in view of the current consumer concerns around sugar. The evidence for the impact of a high-salt diet on increasing blood pressure and therefore cardiovascular disease risk is robustly supported [3] yet the cardiovascular health implications due to sugar intake are less defined [45]. Using targeted messaging to highlight the harmful impact of salt on health outcomes was identified as a motivator for behaviour change. For example, the UK government used a slug carrying a banner with the message “Salt Kills” in the first stage of their campaign [43,46]. Likewise in Finland, high-salt warning labels were placed on high salt foods from 1993, resulting in both a decrease in salt intake and reformulation of foods to reduce salt [36,47]. While labelling has not been part of the current intervention program, messaging through media continues to be utilised. Messaging from the last *Don’t Trust Your Taste Buds* consumer campaign has been re-emphasised in the current *Unpack the Salt* campaign [42]. As introduced above, the product category reports have been used to engage with consumers and industry. Each product category report through the *Unpack the Salt* campaign has involved a media release and the use of infographics. For example, the report on sausages used a visual of trams stating that “Australians eat 1.1 billion sausages per year, that’s 3.5 trams full of salt” [27]. These media releases and messaging raise consumer awareness and have also led to contact with food industry to support salt reduction in their products, showing the value of hard-hitting messaging in engaging with the food industry.

A strength of this qualitative assessment is that it drew upon the key perceptions of stakeholders associated with the VicSalt Partnership, as this is a “living” project, interview findings have already informed changes to the intervention that will enhance project outcomes. Additionally, conducting such an analysis is important within the larger process evaluation, helping future projects to establish effective interventions [14]. However, there were several limitations. Firstly, the range of people interviewed may not give a representative picture of the VicSalt Partnership’s work. For example only one representative from the food industry was interviewed. While this is the case we consciously interviewed people who were either members of, or who had knowledge of, the VicSalt Partnership, in order to get specific feedback. Secondly, given that interviewees were interviewed by a member of the VicSalt Partnership they may have felt they had to respond to questions in a certain way, adding an element of social desirability bias. On the other hand, the experience of the interviewer allowed for more specific questioning, with further bias in the analysis and reporting of results minimised by a researcher, who was previously unfamiliar with the project, analysing the results.

## 5. Conclusions

In conclusion, stakeholder interviews and qualitative analysis have provided a range of important insights. Important findings include the need for clearer engagement with industry, stronger involvement of government and more strategic and hard-hitting messaging to consumers, while maintaining the positive collaboration of the partnership. Following the interviews these findings have been acted upon to strengthen intervention implementation, for example: multiple media releases on the salt content of processed packaged foods; a “call to action” to the federal government; and a positive case study from industry. The evaluation of the VicSalt Partnership is ongoing and the program is expected to provide a wealth of lessons for state led interventions to reduce salt in Australia and globally.

## Figures and Tables

**Table 1:** Number of key stakeholders interviewed, by organisation type.

Type of Organisation	Number of Stakeholders Interviewed	Members of the VicSalt Partnership
State government or statutory agency	5	5
Non-government organisation	4	3
Research	4	4
Industry	1	-
Total number of interviewees	14	12

**Table 2:** Identified needs, remediation opportunities and actions taken for the Victorian Salt Reduction Partnership.

Identified Needs	Evaluation Dimensions [20]	Remediation Opportunities	Actions Taken
Strengthening of the approach taken with food industry to encourage engagement and reformulation	Effectiveness Adoption	Development of “Pledges” for industry to commit to	Commitment statement received from Unilever [24]
		Showcasing positive food industry and manufacturer examples	Woolworths Reformulation case study released [25]
		Support of reformulation efforts	Release of the *Victorian Salt Reduction Innovation Grants* providing up to $25,000 to help SME’s* reformulate [15]
Engagement and involvement of Government	Context Adoption	A policy ask as a way to raise the profile of salt reduction at the federal government level	*Reducing the pressure on our health and economy*—A call to action, taken to government, released February 2018 [26]
Strengthening of salt-reduction messaging	Context	Strong and consistent messaging, similar to that used in regards to sugar or in the UK salt reduction campaigns	Media releases focusing on the high levels of salt in; bread, cooking sauces, ready meals, dips and crackers and sausages, released between March 2017—March 2018 [27]

* SME’s: small- and medium-sized enterprises.

## Supplementary Materials

The following are available online at http://www.mdpi.com/2072-6643/11/1/184/s1, Table S1: VicSalt Partnership stakeholder interviews, semi-structured questionnaire; Table S2: Key themes against the VicSalt Partnership intervention domains.
