# Supplementary material for: Understanding Barriers and Enablers to State Action on Salt: Analysis of Stakeholder Perceptions of the VicHealth Salt Reduction Partnership
Source: Nutrients. 2019 Jan 16;11(1):184. doi: 10.3390/nu11010184 (PMC6356996; doi:10.3390/nu11010184)
Supplement: Supplementary file 1 [file nutrients-11-00184-s001.pdf]

Table S1: VicSalt Partnership stakeholder interviews, semi-structured questionnaire.

**Area**	**Question**	**Sub-questions**
General	Are you aware of the VicHealth Salt Reduction partnership project in Victoria, Australia?	What are the main elements of the intervention?
	Were you or your workplace involved in the development/implementation of VicHealth Salt Reduction Partnership in any way?	If yes, please specify your roles and responsibilities?
		To what extent did you fulfil your roles and responsibilities?
	What organizational issues impacted your capacity to carry out these responsibilities?	
	For each separate element of the intervention (establishing strategic partnerships, policy development and strengthening, generating public debate, innovative approaches with food industry, consumer awareness)	What do you think it is/was trying to achieve?<br>- How will it/did it do this?<br>- Do you think it will/did achieve this? Please give reasons for your answer.
Context	What factors (barriers and enablers) in the Victorian community do you think will /did affect the implementation of the interventions?	
	Who do you think will be/were the main influencers of the program?<br>* *If the intervention has yet to start then finish here. If the intervention has already commenced or has finished then ask the remainder of the questions.*	
Fidelity (Quality of the implementation)	To what extent is/was your role/roles implemented as intended?	What are/were the barriers to full implementation of your roles and responsibilities?
		What other factors affected the implementation of the intervention?

Area	Question	Sub-questions
Dose Delivered (Completeness)	Were all the components of your roles and responsibilities delivered?	What were the barriers to the delivery of your roles and responsibilities?
		What resources were required to undertake your roles?
Dose received	What do you think the community's attitude was overall to the VicHealth Salt Reduction Partnership project?	
	What do you think were the outcomes/impact on the community of establishing strategic partnerships?	
	What do you think were the outcomes/impact on the community of policy development and strengthening?	
	What do you think were the outcomes/impact on the community of generating public debate?	
	Do you think the community was influenced by the consumer awareness campaign? Please give reasons why you think this	What did you think of the consumer awareness campaign? (*Prompt: why/why not?*)
		Do you think the community was influenced by the campaign materials (websites, blog, digital media)? Please give reasons why you think this is.
	What do you think were the outcomes/impact of engagement with the food industry to reduce salt levels in foods? Please give reasons why you think this	Do you think the food industry work influenced the food industry? (*Prompt: why/why not?*)
		Do you think the food industry work had an impact on the community?

Area	Question	Sub-questions
		What impact do you think it had?
Reach	Do you think that the Vichealth Salt Reduction partnership project had adequate reach?	
	Are/were the right stakeholders and community organisations involved?	
	In your opinion, what percentage (approximately) of the Victorian community are aware of VicHealth Salt Reduction Partnership project?	
	In your opinion, what percentage (approximately) of the Victorian community saw the consumer awareness campaign (website, blogs, and digital media)?	
	In your opinion, what proportion of the food industry were engaged in meetings about lowering salt?	

Table S2. Key themes against the VicSalt Partnership intervention domains.

Action Areas	Theme	Quotes
Establishing strategic partnerships (77 quotes)	*Enabling*	*I think the partnership is very strong, we've got a committed group of government and non-government organisations who are leaders not only in Victoria but across Australia in salt reduction, and I think there has been good commitment and a partnership developed around what needs to happen and a call for action for both the community, industry and government as well* (SGSA, member)
		I don't think any of us working in isolation would be able to command that voice. Its amplified having us all doing it together (SGSA, member)
	Barriers	*I feel like with the number of people that have signed onto the state of salt we could have greater engagement across all of these organisations* (SGSA, member)
		I think we should definitely get the other organs involved… we haven't really engaged them well on either the campaign or industry work, or perhaps even the advocacy, so there's got to be a bit of time spent on giving them some ideas on how they can be involved (NGO, member)
Generating Public Debate (22 quotes)	*Approach*	*It's a bit like sugar, whether its evidence based or not we have seen this massive move, the public have absolutely driven it, they've read the books and whether they've said it right or wrong it has actually driven the sugar stuff, not the academics, it's this public opinion that has got out there that sugar is poison which has done that* (NGO, member)
		I think it's got to be something that makes people sit up and take notice and I think the sugar movement has done that really well, almost to the detriment of salt… They have forgotten that salt is actually dangerous and bad for health so yea (NGO, member)
	Barriers	*I think it's trying to bring it [salt] back onto peoples radar… you know it's a very crowded space* (SGSA, member)
Raising consumer awareness (62 quotes)	*Approach*	*It did look like that first campaign had some resonance and had some good response and metrics around it [for consumer awareness] and I think, I kind of feel like that is great but it's a short campaign so I feel like what is really needed here is a really nice commitment to a longer term campaign* (SGSA, member)
	Barriers	*... there's not the understanding of the impact that excessive salt has in the diet, maybe it is because these things, in public health terms, tend to go in cycles and it's just dropped the ball on salt for too long and people don't have that depth of understanding.* (NGO, non-member)
		There is obviously something in the narrative that just doesn't resonate which I think collectively it's our job to change that… salts just not on anyone's radar I don't think (SGSA, member)
Innovative approaches with food industry (114 quotes)	*Approach*	*… having a menu of things that you can positively engage… you know even if we only get them to take on two pledges out of ten at least it is positive change… so if there is positive opportunity to take on a pledge, to be recognised for that and have branding around it and credibility by association, then I think we will see a lot of success* (Industry, non-member)

Action Areas	Theme	Quotes
		I think in terms of getting industry on side and doing things, I think it's just that we've got to have ways of going back to them, building relationships, talking to them, showing them stuff, getting feedback, positive and the negative. It really is the more time and effort that we can put into that the more we're likely to get out (Research, member)
		I know the partnership is very keen on getting salt down, but I think it's not reasonable to look at salt without looking at the other products, if you are talking to food industry because then you are only doing a third of the job, or you could do a whole lot more to get a product better, if you used traffic lights it would get a higher rating, or more stars (SGSA, member)
		… highlighting to industry that the consumers do want a healthier food supply and it's a great marketing opportunity. That I don't think the industry has really embraced and they don't really see the potential, so I think there is scope there for opening their eyes to that, so then they will take action because it will be to their advantage (Research, member)
	Barriers	*… getting companies to change how much salt they have in things, how they market things, how they monitor salt is going to be challenging, more interesting things in this space for them at the moment; not least sugar, not least health star rating...* (Research, member)
		… the time that it takes to engage industry and develop relationships to achieve progress seems to be a much slower pace than we anticipated, again just getting it on the radar of industry again (SGSA, member)
		Being able to harness it at the state level is always a bit problematic because it breaks the style across the country (NGO, non-member)
	Potential Enablers	*I think the health stars gives us something to focus on, of all the things that we've talked about with industry over the last decade, that's the one thing where they have genuinely been interested and there has been real movement and I think it is because they've seen a commercial opportunity there...*(Research, member)
		I think definitely the partnership with industry, I think the approach we are on now with what's been achieved, so who across industry has been able to reduce their sodium content without adverse community reactions to lower salt is a really good approach to try encourage others, to say it can be done and it has been done (SGSA, member)
		Victorian government has been in the healthy eating space at a pretty consistent effort and they are also kind of in the food industry space as well, they've got the support through their healthy eating advisory service so that's a real enabler I think (SGSA, member)
Strengthen healthy policies (35 quotes)	*Approach*	*Well I think getting industry to agree to some targets or the government, we should work towards it and someway of monitoring it and accountability for it, any commitment. How you get that I don't actually know* (Research, member)
	Enablers	*I think it is positive that the Victorian department of health is part of the group and they are clearly looking at ways to integrate this into broader health and diet/nutrition programs, which is good. Whether we've really achieved everything that we set out, I don't know to be honest.* (Research, member)
		… the best level it [salt reduction] is done is at a national level but agitation and work at the state level I think is going to be incredibly helpful, I think that as Victoria has this underway it will strengthen the arm of advocates in Canberra (NGO, non-member)

Action Areas	Theme	Quotes
	Barriers	*Victorian policy is a much broader healthy eating policy and healthy eating guidelines really, I think it's more about the fact the salt reduction is part of that and inherent within it but it is not the focus…* (SGSA, member)

*SGSA: interviewee from state government or a statutory agency.
